# Semi-parametric validation of genomic predictions and polygenic risk scores with the Blupf90 software suite

**DOI:** 10.1093/g3journal/jkaf136

**Published:** 2025-06-12

**Authors:** Matias Bermann, Alejandra Álvarez Múnera, Ignacy Misztal, Daniela Lourenco

**Affiliations:** Department of Animal and Dairy Science, University of Georgia, Athens, GA 30602, USA; Department of Animal and Dairy Science, University of Georgia, Athens, GA 30602, USA; Department of Animal and Dairy Science, University of Georgia, Athens, GA 30602, USA; Department of Animal and Dairy Science, University of Georgia, Athens, GA 30602, USA

**Keywords:** validation, accuracy, genomic predictions, polygenic risk scores

## Abstract

Validation of genomic predictions or polygenic risk scores is key for model selection and evaluating the performance of the chosen prediction machinery. Non-parametric validation, such as cross-validation, is popular but does not account for population structure and the fact that the interest could be in validating a set of individuals and not the entire population. Semi-parametric methods, such as the LR method, also use removed records to validate predictions, account for population structure, and allow focus on a specific set of individuals of interest. Confidence intervals are obtained using semi-parametric methods without the need for repeated cross-validation. We developed a tool within the Blupf90 software suite, called validationf90, that allows researchers to conduct semi-parametric validation from the solutions obtained from that software suite. validationf90 calculates different validation statistics and their confidence intervals for a pre-defined set of individuals of interest, reflecting the bias and accuracy of genomic predictions. The program allows for genomic predictions obtained from frequentist and Bayesian methods, as well as for categorical data. validationf90 can validate any model supported by the Blupf90 software suite and can be used with animal, plant, and human datasets. Predictions obtained with other software can be provided to validationf90 as long as the input format matches with the Blupf90 format.

## Teaser

Validation of genomic predictions or polygenic risk scores is crucial for model selection and validating the performance of the chosen model. Non-parametric cross-validation does not consider population structure, and that not all individuals are of the same interest for validation. Semi-parametric validation overcomes these issues. Bermann *et al*. developed a software called validationf90, which performs semi-parametric validation within the Blupf90 software suite. This type of validation, as well as the software tool, is applicable to animal, plant, and human datasets.

## Introduction

Validation of genomic predictions and polygenic risk scores is crucial for model selection and assessing the performance of the current prediction machinery. Statistical validation could be parametric (e.g. Rencher and Schaalje 2008), semi-parametric (Thompson 2001; Legarra *et al*. 2008; Legarra and Reverter 2018), or non-parametric (Hastie *et al*. 2009). Parametric validation consists of statistics calculated from evaluating the likelihood, such as the logarithm of the likelihood, Akaike Information Criterion (AIC), or Bayesian Information Criterion (BIC), or as closed-form functions of estimators such as Mallows' Cp, R2 from linear regression, receiver operating characteristic (ROC) curve, among others. Although parametric validation could be used for genomic prediction models, it gives emphasis on the fitting of the model to the data, whereas the goal of genomic prediction is to have high accuracy and low biased estimated breeding values, marker effects, or polygenic risk scores (Thompson 2001; Daetwyler *et al*. 2013). Non-parametric validation comprises cross-validation (CV) and bootstrapping (Efron and Tibshirani 1993). On the one hand, CV and its variants like stratified CV or k-fold CV consist of splitting the data into training and testing sets and predicting the testing set using predictions obtained from the training set (Hastie *et al*. 2009). On the other hand, bootstrapping works by sampling with replacement testing and training datasets. Then, in both cases, statistics like correlations and slopes of regressions are obtained by comparing predictions and observations. Non-parametric validation is popular for evaluating genomic predictions due to its simplicity and because some statistics resemble genetic parameters, such as the accuracy of estimated breeding values. However, it does not consider the distributional properties of the genomic predictions (Henderson 1975) and does not directly consider population structure (Thompson 2001; Daetwyler *et al*. 2013). Legarra and Reverter (2018) developed a semi-parametric method, named the LR method, for validating genomic predictions while considering their distributional properties. The LR method is similar to CV but compares predictions from the complete (whole) dataset against those from a reduced (partial) dataset and provides expected values of those statistics. Thanks to its simplicity and because it considers population structure, the LR method is widely spread in the animal breeding field (e.g. Vallejo *et al*. 2019; Bermann *et al*. 2021; Massender *et al*. 2022; Aliakbari *et al.*, 2022; Lázaro *et al.*, 2024) and is gaining importance in the plant breeding field (e.g. Bornhofen *et al*. 2022; Callister *et al*. 2022; Guo *et al*. 2023; Tessema *et al*. 2024; Raffo *et al*. 2025).

The Animal Breeding and Genetics group at the University of Georgia has been actively conducting research on genomic selection and genetic prediction methods (https://nce.ads.uga.edu). Many of the methods developed by the group were programmed in the Blupf90 software suite (Lourenco *et al*. 2022), which is available for researchers and companies and has an average of 2,000 monthly downloads. The Blupf90 software suite is programmed in Fortran and works with executable binaries and plain text parameter files. The software supports genomic information, has great flexibility for statistical modeling, and is programmed to maximize computing efficiency.

Validation statistics from the LR method can be easily calculated with the output files provided by some of the programs of the Blupf90 software suite. As they are computed from random variables, validation statistics have associated incertitude. Therefore, inference on those validation statistics should account for their point estimate and standard error. Although the LR method validation statistics could be obtained directly from the genomic predictions, their standard error is hard to calculate (Bermann *et al*. 2024), making proper statistical inference unfeasible. Therefore, we developed a software called validationf90, which helps the user conduct a proper semi-parametric validation by obtaining the LR method statistics and their standard errors. This article aims to show how to validate genomic predictions within the Blupf90 software suite with validationf90 and introduce its main functionalities.

## Materials and methods

### The LR method

For genomic predictions, we consider a linear mixed model of the following form:


(1) y=Xb+Zu+Wp+e,

where y is the vector of phenotypes, b is the vector of fixed effects, u is the vector of additive genetic effects, p is the vector of random effects except for the additive genetic effect, e is the vector of errors, and X, Z, and W are incidence matrices.

The LR method creates a subset of the whole dataset called *partial* dataset by removing observations for the testing or focal set. Genomic predictions obtained with the whole dataset are denoted by ${\hat{u}}_{w}$, whereas genomic predictions obtained from the partial dataset are denoted by ${\hat{u}}_{p}$. Based on Henderson (1975), the joint distribution of ${\hat{u}}_{w}$ and ${\hat{u}}_{p}$ assuming that selection is properly taken into account:


(2) $$\left[\begin{array}{c} {\hat{u}}_{w}\\ {\hat{u}}_{p} \end{array}\right]∼\mathrm{MVN}\left(\left[\begin{array}{c} 0\\ 0 \end{array}\right],\left[\begin{array}{cc} G-C_{w}^{22} & G-C_{p}^{22}\\ G-C_{p}^{22} & G-C_{p}^{22} \end{array}\right]\right),$$

where G is the variance of u. Depending on the application, G could be the additive relationship matrix, a genomic relationship matrix (e.g. VanRaden 2008; Hayes *et al*. 2009; Yang *et al*. 2010), a single-step genomic relationship matrix (Legarra *et al*. 2009), or a kernel-based relationship matrix (Morota and Gianola 2014).

The validation statistics of the LR method are:

- Bias: the mean difference between ${\hat{u}}_{w}$ and ${\hat{u}}_{p}$, which has an expected value of zero if the predictions are unbiased. In the presence of bias, there is a wrong estimation of the genetic trend.
- Dispersion or inflation: the slope of the regression of ${\hat{u}}_{w}$ on ${\hat{u}}_{p}$, which has an expected value of one if there is no over/under estimation of ${\hat{u}}_{p}$. Values lower than one indicate that predictions are overestimated or inflated, while values greater than one indicate that predictions are underestimated or deflated.
- Ratio of accuracies: the Pearson correlation coefficient between ${\hat{u}}_{w}$ and ${\hat{u}}_{p}$, which reflects the increase in accuracy when adding observations to the partial data.
- Reliability: the covariance between and ${\hat{u}}_{w}$ and ${\hat{u}}_{p}$, divided by the variance of the focal individuals, which reflects the squared correlation between true and estimated breeding values. The square root of the reliability is the accuracy, that is, the correlation between true and estimated breeding values.
- Predictivity: the correlation between ${\hat{u}}_{p}$ and adjusted phenotypes $\left(y^{*}\right)$, divided by the square root of the heritability, which reflects the accuracy of estimated breeding values.

Statistical inference for the validation statistics is carried out by $CI_{100(1-\alpha )}(\theta )=\theta \pm z_{1-\frac{\alpha }{2}}\;sd(\theta )$ (Wald 1943), where CI is the confidence interval, *θ* is the validation statistic, $z_{1-\frac{\alpha }{2}}$ is the value of the standard normal distribution quantile function for the confidence level $1-\frac{\alpha }{2}$, α is the significance level, and sd(θ) is the asymptotic standard error of *θ*.

The mathematical expressions for the validation statistics were derived in Legarra and Reverter (2018), whereas Bermann *et al*. (2024) provided formulae for their asymptotic variances. Details are provided in Table 1.

**Table 1.** Mathematical expression for the validation statistics and their exact and approximated confidence intervals.

	Statistic	Exact confidence intervals	Approximated confidence intervals
Bias	$\mu_{wp}=mean({\hat{u}}_{p}-{\hat{u}}_{w})$	$\mathrm{CI}(\mu_{wp})=\mu_{wp}\pm z_{1-\;\frac{\alpha }{2}}\;\sqrt{n^{-2}1^{\prime }(C_{p}^{22}-C_{w}^{22})1}$	$\mathrm{CI}(\mu_{wp})\approx \mu_{wp}\pm z_{1-\;\frac{\alpha }{2}}\;\sqrt{\frac{\sigma_{g}^{2}}{n}({\bar{rel}}_{w}-{\bar{rel}}_{p})}$
Dispersion	$b_{wp}=\frac{cov({\hat{u}}_{p},{\hat{u}}_{w})}{var({\hat{u}}_{p})}$	$\mathrm{CI}(b_{wp})=b_{wp}\pm z_{1-\;\frac{\alpha }{2}}\;\sqrt{\frac{\mathrm{tr}(S(C_{p}^{22}-C_{w}^{22})S(G-C_{p}^{22}))}{2\;\mathrm{tr}(S(G-C_{p}^{22})S(G-C_{p}^{22}))+\mathrm{tr}{\left(S(G-C_{p}^{22})\right)}^{2}}}$	$\mathrm{CI}(b_{wp})\approx b_{wp}\pm z_{1-\;\frac{\alpha }{2}}\;\sqrt{\frac{\left(c-1)(var(rel_{p})+{\bar{rel}}_{p}^{2}\right)}{2(var(rel_{p})+{\bar{rel}}_{p}^{2})+n\;{\bar{rel}}_{p}^{2}}}$
Ratio of accuracies	$\rho_{wp}=corr({\hat{u}}_{p},{\hat{u}}_{w})$	$\mathrm{CI}(\rho_{wp})=\tanh\;\left(\tanh^{-1}(\rho_{wp})\pm z_{1-\;\frac{\alpha }{2}}\frac{1}{\sqrt{n-3}}\right)$	$\mathrm{CI}(\rho_{wp})=\tanh\;\left(\tanh^{-1}(\rho_{wp})\pm z_{1-\;\frac{\alpha }{2}}\frac{1}{\sqrt{n-3}}\right)$
Reliability	$\rho_{cov_{wp}}^{2}=\frac{cov({\hat{u}}_{p},{\hat{u}}_{w})}{\sigma_{g_{i}}^{2}}$	$\mathrm{CI}(\rho_{cov_{wp}}^{2})=\rho_{cov_{wp}}^{2}\pm z_{1-\;\frac{\alpha }{2}}\;\sqrt{\frac{\mathrm{tr}(S(C_{p}^{22}-C_{w}^{22})S(G-C_{p}^{22}))+2\;\mathrm{tr}(S(G-C_{p}^{22})S(G-C_{p}^{22}))}{n\;\sigma_{g_{i}}^{2}}}$	$\mathrm{CI}(\rho_{cov_{wp}}^{2})\approx \rho_{cov_{wp}}^{2}\pm z_{1-\;\frac{\alpha }{2}}\;\sqrt{\frac{(1+c)\sigma_{g}^{4}}{n\;\sigma_{g_{i}}^{4}}(var(rel_{p})+{\bar{rel}}_{p}^{2})}$
Predictivity	$\rho_{y^{*},{\hat{u}}_{p}}=\frac{corr(y^{*},{\hat{u}}_{p})}{h}$	$\mathrm{CI}(\rho_{y^{*},{\hat{u}}_{p}})=\frac{1}{h}\;\tanh\;\left(h\;\tanh^{-1}(\rho_{y^{*},{\hat{u}}_{p}})\pm z_{1-\;\frac{\alpha }{2}}\frac{1}{\sqrt{n-3}}\right)$	$\mathrm{CI}(\rho_{y^{*},{\hat{u}}_{p}})=\frac{1}{h}\;\tanh\;\left(h\;\tanh^{-1}(\rho_{y^{*},{\hat{u}}_{p}})\pm z_{1-\;\frac{\alpha }{2}}\frac{1}{\sqrt{n-3}}\right)$

*mean*, sample mean; *cov*, sample covariance; *var*, sample variance; *corr*, sample correlation coefficient; $\sigma_{g_{i}}^{2}$, genetic variance of the focal or testing individuals; $y^{*}$, adjusted phenotypes; *h*, square root of the heritability; $C_{p}^{22}$, prediction error (co)variance for the focal set obtained with the partial dataset; $C_{w}^{22}$, prediction error (co)variance for the focal set obtained with the complete dataset; G, genetic (co)variance for the focal set; $S=I-n^{-1}11^{\prime }$; tanh, hyperbolic tangent; $rel_{w}$, reliabilities for the focal set obtained with the complete dataset; $rel_{p}$, reliabilities for the focal set obtained with the partial dataset; ${\bar{rel}}_{w}$, average of $rel_{w}$; ${\bar{rel}}_{p}$, average of $rel_{p}$; $\sigma_{g}^{2}$, genetic variance.

### Main functionalities

Binaries of validationf90 for different operative systems are available at https://nce.ads.uga.edu/wiki/doku.php?id=distribution. As with many of the programs in the Blupf90 software suite, validationf90 works with a parameter file and specific options to manage its behavior. The program will calculate validation statistics and, optionally, their standard errors and 95% confidence intervals for a random effect (usually, the genetic effect but could be maternal or permanent environmental effect) specified by the user and for all the traits available in the model. validationf90 is activated with OPTION validation eff list_1 list_2 … list_n, where eff is the effect number for which validation will be performed and list_1 list_2 … list_n are files containing the identification of the individuals in the focal or testing set. The number of files with identifications could be one (the same testing group is used for all the traits) or equal to the number of traits (each trait has its own testing group).

The program automatically reads two solution files generated by two different runs of blupf90+, one with the whole and the other with the partial dataset, and extracts ${\hat{u}}_{w}$ and ${\hat{u}}_{p}$ for the focal set. The two different runs should be done by the user, while the extraction of ${\hat{u}}_{w}$ and ${\hat{u}}_{p}$ for the focal set is done internally by validationf90. The default names for the solutions files are solutions_whole and solutions_partial for the whole and partial datasets, respectively. The prefix of the solutions files can be changed by an option as long as the suffixes _whole and _partial are maintained. Solutions from other statistical packages can be provided to validationf90, as long as the input format matches the Blupf90 solution file format and a parameter file is provided.

After reading the solutions, the program will calculate the trait's heritabilities based on the covariance matrices read in the parameter file. These heritabilities will be used to calculate the predictivity and the variance of the focal individuals to obtain the reliability estimator (see Table 1). The user can also provide the variance of the focal set.

validationf90 calculates bias, dispersion, ratio of accuracies, and reliability by default. The user can add OPTION predictive_ability to the parameter file to force the program to calculate the predictivity (i.e. predictive ability) for each trait based on the file yhat_residual, which should be obtained beforehand using the program predictf90.

By default, the program will not calculate standard errors and confidence intervals. For activating the calculation, the user should add OPTION se mode to the parameter file, where mode could be either exact for exact asymptotic variances and confidence intervals (see column 3 in Table 1), approx for approximated asymptotic variances (see column 4 in Table 1), or boot for variances calculated by bootstrapping (Efron and Tibshirani 1993). Users may need to add different options when running blupf90+, depending on the chosen mode.

### *Running*  validationf90

Henceforth, eff will refer to the effect number to validate, n will refer to the number of traits, and ids_i will refer to a file containing a column of validation individual's identification for the *i*th trait; if no subscript is provided, it is assumed that the same list holds for all the traits.

The main steps for conducting a validation with validationf90 are as follows:

1. Run renumf90 to renumber the whole dataset.
2. Create the partial dataset by subsetting the renumbered data from the first step.
3. Run blupf90+ with the whole dataset. The predictions will be in the file solutions for versions from blupf90+ equal to or less than 2.60, which should be renamed to solutions_whole right after the program finishes. For versions equal to or greater than 2.61, the user might choose to change the solutions' file name with OPTION solfile solutions_whole before running the program or run the program without that option and proceed as for versions equal to or less than 2.60.
4. Run blupf90+ with the partial dataset. The solutions' file should be named solutions_partial.
5. Run validationf90 by adding OPTION validation eff ids_1 ids_2 … ids_n if each trait has a different testing set or OPTION validation eff ids, otherwise.

Steps 1 and 2 are common for all ways of running validationf90; therefore, they will be omitted from the following descriptions. The same holds for OPTION validation eff ids_1 ids_2 … ids_n.

To include the calculation of predictive ability, the user should run the following sequence of programs:

1. Run blupf90+ with the whole dataset to obtain solutions_whole.
2. Run predictf90 with OPTION include_effects eff and OPTION solfile solutions_whole.
3. Run blupf90+ with the partial dataset to obtain solutions_partial.
4. Run validationf90 with OPTION predictive_ability.

By running validationf90 as shown, the user will obtain only validation statistics but not confidence intervals. Although the option for calculating predictivity will be omitted from the following sequences, the user can add it as previously explained.

For calculating confidence intervals by bootstrap, the user should:

1. Run blupf90+ with the whole dataset to obtain solutions_whole.
2. Run blupf90+ with the partial dataset to obtain solutions_partial.
3. Run validationf90 with OPTION se boot.

The number of bootstrap samples is 10,000 by default. To modify it, the user can add OPTION nrepboot x to the parameter file of validationf90, where x is the desired number of bootstrap samples.

For obtaining confidence intervals based on exact asymptotic variances, the sequence should be:

1. Run blupf90+ with the whole dataset to obtain solutions_whole. Add OPTION store_pev_pec eff full to the parameter file. This will create n binary files named ebv_pev_i_1, i  =  1, …, n, which the user should rename them as ebv_pev_i_1_whole.
2. Run blupf90+ with the partial dataset to obtain solutions_partial and add OPTION store_pev_pec eff full to the parameter file. As before, the user should rename ebv_pev_i_1 as ebv_pev_i_1_partial.
3. Run validationf90 with OPTION se exact.

It is worth noticing that calculating exact asymptotic variance requires inverting the mixed model equations; therefore, it could be computationally costly. Inverting the mixed model equations is done internally in blupf90+ when OPTION store_pev_pec eff full is included in the parameter file.

Finally, obtaining confidence intervals based on approximated asymptotic variances requires approximating accuracies with accf90GS2 or accf90GS3 (further named indistinctly as accf90GSx), which are available under a research agreement. Although blupf90+ can calculate accuracies, its computational cost is equal to inverting the mixed model equations. Hence, one would use confidence intervals based on exact asymptotic variances. In any case, the steps for obtaining confidence intervals based on approximated asymptotic variances are:

1. Obtain solutions for the whole dataset and run accf90GSx. Rename the output sol_and_acc by sol_and_acc_whole.
2. Repeat the previous step with the partial dataset to obtain sol_and_acc_partial.
3. Run validationf90 with OPTION se approx and OPTION prefix sol_and_acc.

Details for the optional arguments for validationf90 are provided on the website (https://nce.ads.uga.edu/wiki/doku.php?id=readme.validationf90). Details on how to run renumf90, blupf90+ and other programs form the Blupf90 software suite are in Lourenco *et al*. (2020).

### Application example

We used a simulated dairy cattle dataset, which can be found in https://github.com/masuday/data/tree/master/tutorial/rawfiles. These data consist of a simulated dairy cattle population with a pedigree of 4,641 animals spanning 11 generations. Four traits were simulated as the sum of an overall mean, generation, and sex as fixed effects, and the additive genetic effect and residual as random effects. Semi-parametric validation was carried out on the last generation, which had 461 animals. The parameter files for the full pipeline are in Supplementary File.

## Results and discussion

**Fig. 1.**
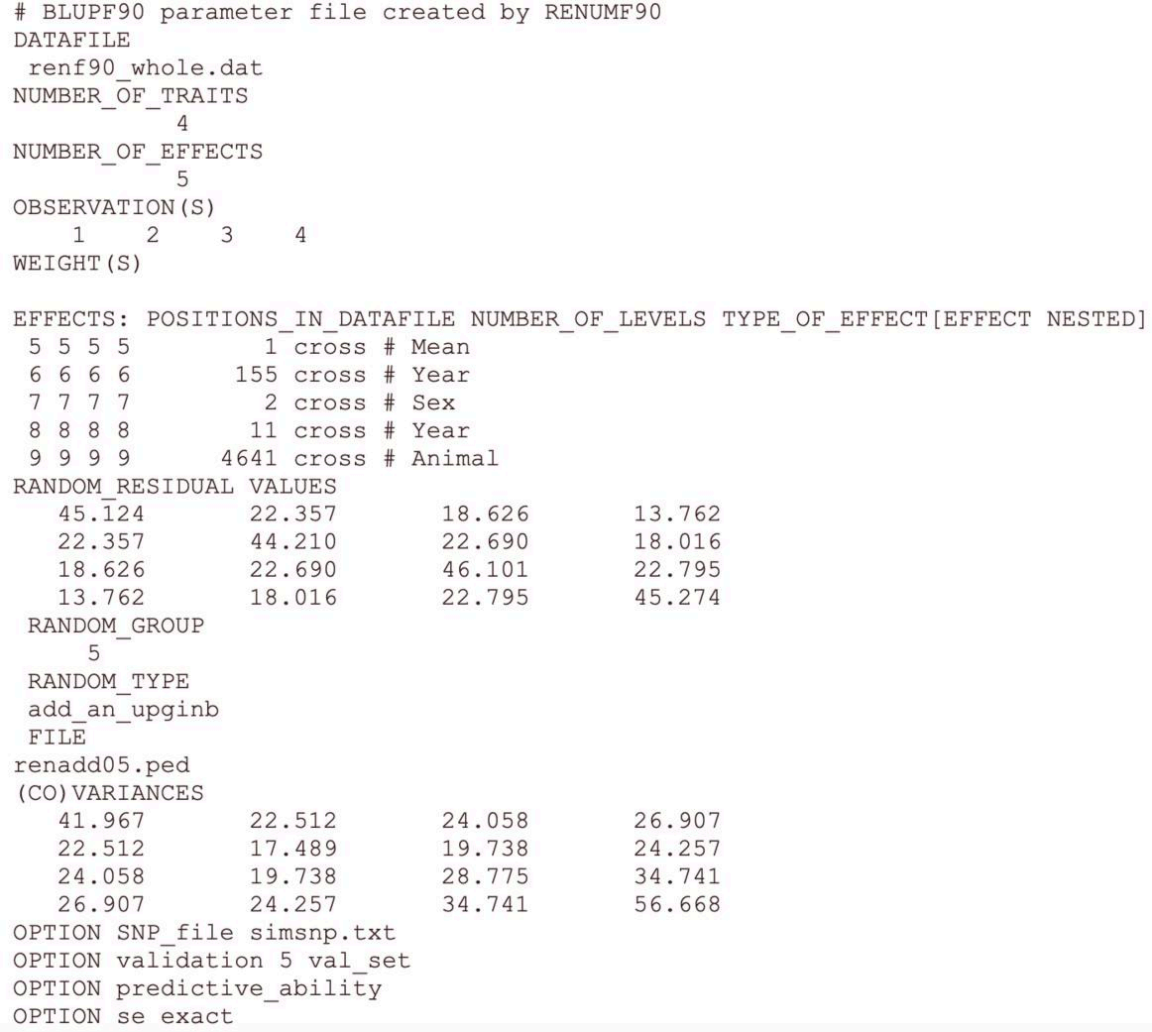
Parameter file of validationf90 for the simulated dataset.

**Fig. 2.**
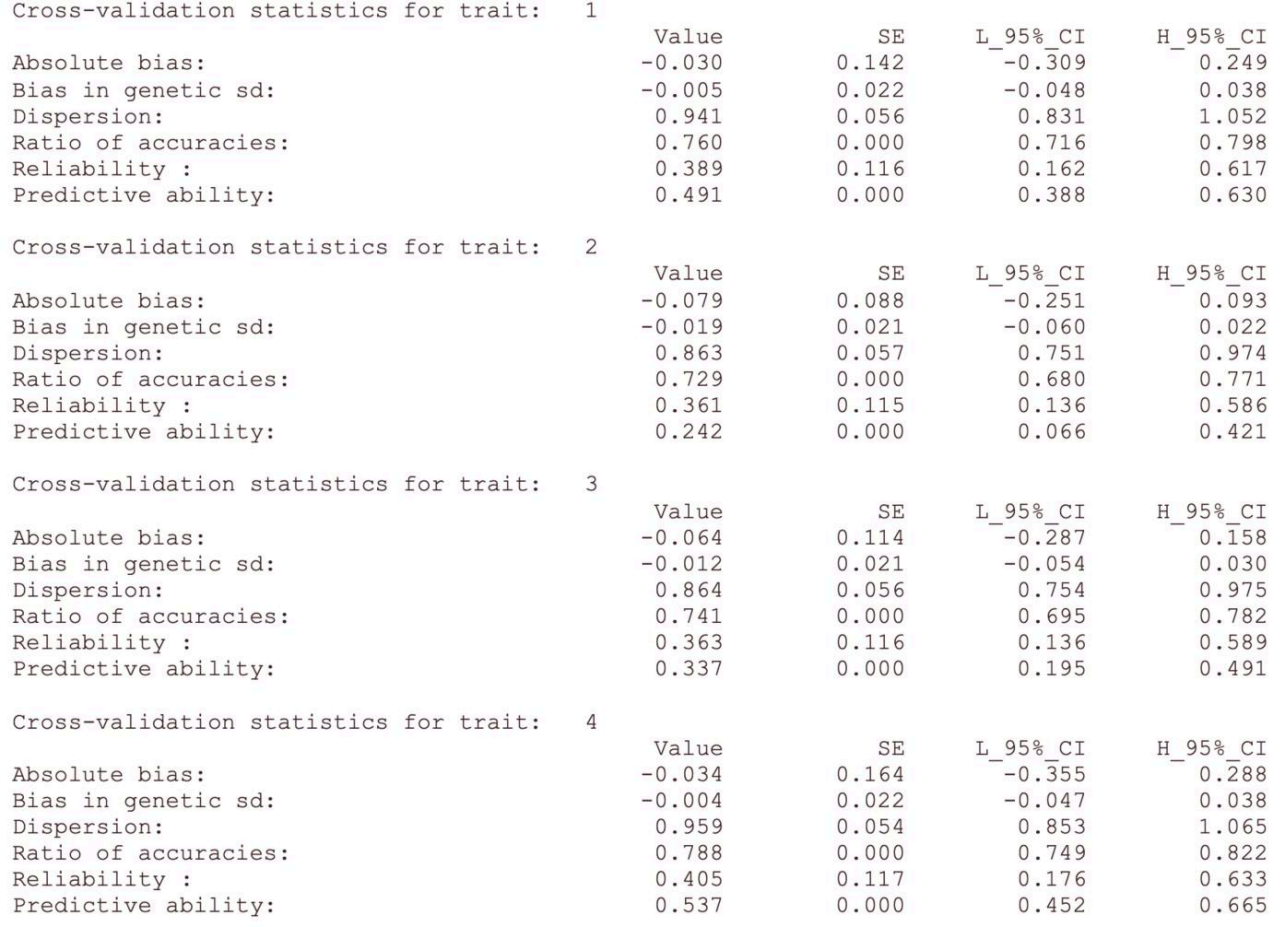
Output validationf90 for the simulated dataset. Point estimate, standard errors (SE), and 95% confidence intervals are provided.

The parameter file for validationf90 for the simulated dataset can be seen in Fig. 1, while the results for the validation statistics in Fig. 2. The full pipeline for replicating the analysis can be found in Supplementary File. Due to the small size of the data, we chose to use exact confidence intervals. The values of the validation statistics lie within their expected values; however, some of the confidence intervals are large due to the small number of validation individuals. In theory, the values of the reliability should match the squared predictive ability. Disagreements could be explained due to the small sample size, estimates of heritability, and approximation of the variance of focal individuals (Macedo *et al*. 2020).

Validation of genomic predictions is essential for model selection and assessing the chosen model's performance for genetic and genomic predictions. Prediction of the genetic merit and polygenic risk scores involves unbalanced datasets, the presence of population structure, and uneven importance of individuals for prediction purposes (Daetwyler *et al*. 2013). In such cases, conducting non-parametric validations such as k-fold cross-validation is unsuitable for evaluating the quality of genomic predictions (Legarra and Reverter 2018). For instance, genomic predictions for dairy cattle involve pedigrees that trace back to 1910; however, the interest is in predicting the genetic merit of young selection candidates. In such a case, one would want to calculate the accuracy of predictions for those young selection candidates while overlooking older animals in the pedigree. Thus, where the population cannot be split randomly or where there is only one way to subset the data for validation, semi-parametric validation, such as the LR method, provides the proper statistical framework for evaluating genomic models. Considering the variation of validation statistics is crucial for comparing methods and avoiding concluding that there exist differences between models or methods where there are none. In other words, considering confidence intervals of validation statistics reduces the probability of incurring in type one errors. In a previous study (Bermann *et al*. 2024), we showed the importance of considering genomic predictions as random variables when calculating validation statistics such as those presented in this study. If confidence intervals for validation statistics were calculated as if genomic predictions were fixed (i.e. using known formulas for simple linear regression), the confidence intervals would be narrower than they should be. Therefore, researchers could conclude that significant differences exist where there are not. Semi-parametric validation with proper confidence intervals (Bermann *et al*. 2024) helps avoid such a problem. Since calculating confidence intervals for semi-parametric validation statistics is not an easy task, validationf90 helps researchers properly validate genetic and genomic predictions.

Designing validationf90 as a separate program allows the users to fully customize and run their analysis in steps, ensuring reliable results and a robust pipeline. The software is designed to efficiently read solution files given by blupf90+, making it possible to process the genomic predictions of a few focal individuals from a solution file of hundreds of millions of lines. This design also allows the researchers to use different methods for predicting the genetic merit, such as Bayesian methods, and conduct validation for categorical traits (Bermann *et al*. 2021). For example; Albiñana *et al*. (2023) investigated the increase in predictive accuracy for different psychiatry phenotypes, such as attention-deficit/hyperactivity disorder, bipolar disorder, and schizophrenia, among others, by moving from a single to a multiple-trait prediction scenario. They calculated what they called multi-polygenic risk scores from publicly available GWAS using generalized linear models, tree gradient boosting, and SNP-BLUP. The authors performed a 5-fold cross-validation by adjusted variance explained in the liability scale. Using the Blupf90 software suite, studies like those from Albiñana *et al*. (2023) could also calculate polygenic risk scores from threshold models using gibbsf90+ and perform a semi-parametric validation with validationf90. Mahjani *et al.* (2020) investigated the presence of maternal effects on the risk for obsessive-compulsive disorder (OCD). In their study, they applied a threshold model with the Blupf90 software suite and concluded that there is a presence of maternal effects on OCD based on the fact that the maternal variance of their model was significantly different than zero. Possible further steps of their study could include validating the maternal effect estimates using validationf90.

Recently, many studies compared genomic predictions obtained from linear mixed models vs those obtained from machine learning methods (e.g. John *et al*. 2022; Lee *et al.*, 2023; Li *et al*. 2024). In most studies, the criterion for comparison is the correlation between estimated breeding values and adjusted phenotypes or predicted phenotypes and observed phenotypes, while the bias of the predictions is usually overlooked. In any case, inference on those validation statistics is generally done by *k*-fold validation, which overlooks the covariance structure among the testing individuals and could underestimate the variation of the validation statistics (Bermann *et al*. 2024). In such cases, we recommend performing a semi-parametric validation for the predictions obtained from linear mixed models, which possibly would provide more accurate confidence intervals and change the conclusion of the comparison between genomic predictions obtained either from linear mixed models or from machine learning methods.

The LR method validation statistics are conditional on the underlying statistical model, hence, on the chosen covariance matrix for the estimated breeding values. Thus, if the model or the covariance matrix is improper, inference with the LR method could be misleading. All the programs from the Blupf90 software suite allow the user to choose between three different definitions (Leutenegger *et al.*, 2003; Amin *et al*. 2007; VanRaden 2008; Yang *et al*. 2010) and scalings (VanRaden 2008; Hayes *et al*. 2009; Gianola *et al*. 2009) of the genomic relationship matrix. Furthermore, the user can provide the entire covariance matrix for random effects from an external file. Thanks to these functionalities, the user has full control of the predictions and their validation.

## Conclusions

validationf90 is a computing tool integrated into the Blupf90 software suite that allows users to conduct proper semi-parametric validation of genomic predictions or polygenic risk scores. The program gives estimates and confidence intervals of many validation statistics, reflecting the bias and accuracy of predictions. validationf90 accepts outputs from frequentist and Bayesian methods, as well as categorical data. Coupled with high computational efficiency, validationf90 is suitable for performing validation of predictions of any animal, plant, or human genomic datasets.

## Supplementary Material

jkaf136_Supplementary_Data

## Data Availability

The data is available at https://github.com/masuday/data/tree/master/tutorial/rawfiles. Binaries for different operative systems are available at https://nce.ads.uga.edu/wiki/doku.php?id=distribution. The parameter files to run the full pipeline are provided in the Supplementary File. Supplemental material available at G3 online.
