# Supplementary material for: Semi-parametric validation of genomic predictions and polygenic risk scores with the Blupf90 software suite
Source: G3 (Bethesda). 2025 Jun 12;15(8):jkaf136. doi: 10.1093/g3journal/jkaf136 (PMC12341872; doi:10.1093/g3journal/jkaf136)
Supplement: jkaf136_Supplementary_Data [file jkaf136_supplementary_data.docx]

Parameter file for renumf90

# parameter file for renumf90

DATAFILE

simdata.txt

TRAITS

9 10 11 12

FIELDS_PASSED TO OUTPUT

WEIGHT(S)

RESIDUAL_VARIANCE

45.124 22.357 18.626 13.762

22.357 44.210 22.690 18.016

18.626 22.690 46.101 22.795

13.762 18.016 22.795 45.274

EFFECT

5 5 5 5 cross alpha # Mean

EFFECT

6 6 6 6 cross alpha # Farm

EFFECT

7 7 7 7 cross alpha # Sex

EFFECT

8 8 8 8 cross alpha # Year

EFFECT

1 1 1 1 cross alpha # Animal

RANDOM

animal

FILE

simped.txt

FILE_POS

1 2 3 0 0

SNP_FILE

simsnp.txt

PED_DEPTH

0

(CO)VARIANCES

41.967 22.512 24.058 26.907

22.512 17.489 19.738 24.257

24.058 19.738 28.775 34.741

26.907 24.257 34.741 56.668

Parameter file for blupf90+ with the whole data

# BLUPF90 parameter file created by RENUMF90

DATAFILE

renf90_whole.dat

NUMBER_OF_TRAITS

4

NUMBER_OF_EFFECTS

5

OBSERVATION(S)

1 2 3 4

WEIGHT(S)

EFFECTS: POSITIONS_IN_DATAFILE NUMBER_OF_LEVELS TYPE_OF_EFFECT[EFFECT NESTED]

5 5 5 5 1 cross # Mean

6 6 6 6 155 cross # Year

7 7 7 7 2 cross # Sex

8 8 8 8 11 cross # Year

9 9 9 9 4641 cross # Animal

RANDOM_RESIDUAL VALUES

45.124 22.357 18.626 13.762

22.357 44.210 22.690 18.016

18.626 22.690 46.101 22.795

13.762 18.016 22.795 45.274

RANDOM_GROUP

5

RANDOM_TYPE

add_an_upginb

FILE

renadd05.ped

(CO)VARIANCES

41.967 22.512 24.058 26.907

22.512 17.489 19.738 24.257

24.058 19.738 28.775 34.741

26.907 24.257 34.741 56.668

OPTION SNP_file simsnp.txt

OPTION store_pev_pec 5 full

OPTION solfile solutions_whole

This will produce the following outputs: ebv_pev_1_1, ebv_pev_2_1, ebv_pev_3_1, and ebv_pev_4_1, which should be renamed as ebv_pev_1_1_whole, ebv_pev_2_1_whole, ebv_pev_3_1_whole, ebv_pev_4_1_whole, respectively.

Parameter file for predictf90

# BLUPF90 parameter file created by RENUMF90

DATAFILE

renf90_whole.dat

NUMBER_OF_TRAITS

4

NUMBER_OF_EFFECTS

5

OBSERVATION(S)

1 2 3 4

WEIGHT(S)

EFFECTS: POSITIONS_IN_DATAFILE NUMBER_OF_LEVELS TYPE_OF_EFFECT[EFFECT NESTED]

5 5 5 5 1 cross # Mean

6 6 6 6 155 cross # Year

7 7 7 7 2 cross # Sex

8 8 8 8 11 cross # Year

9 9 9 9 4641 cross # Animal

RANDOM_RESIDUAL VALUES

45.124 22.357 18.626 13.762

22.357 44.210 22.690 18.016

18.626 22.690 46.101 22.795

13.762 18.016 22.795 45.274

RANDOM_GROUP

5

RANDOM_TYPE

add_an_upginb

FILE

renadd05.ped

(CO)VARIANCES

41.967 22.512 24.058 26.907

22.512 17.489 19.738 24.257

24.058 19.738 28.775 34.741

26.907 24.257 34.741 56.668

OPTION SNP_file simsnp.txt

OPTION include_effects 5

OPTION solfile solutions_whole

Parameter file for blupf90+ with the partial dataset

# BLUPF90 parameter file created by RENUMF90

DATAFILE

renf90_partial.dat

NUMBER_OF_TRAITS

4

NUMBER_OF_EFFECTS

5

OBSERVATION(S)

1 2 3 4

WEIGHT(S)

EFFECTS: POSITIONS_IN_DATAFILE NUMBER_OF_LEVELS TYPE_OF_EFFECT[EFFECT NESTED]

5 5 5 5 1 cross # Mean

6 6 6 6 155 cross # Year

7 7 7 7 2 cross # Sex

8 8 8 8 11 cross # Year

9 9 9 9 4641 cross # Animal

RANDOM_RESIDUAL VALUES

45.124 22.357 18.626 13.762

22.357 44.210 22.690 18.016

18.626 22.690 46.101 22.795

13.762 18.016 22.795 45.274

RANDOM_GROUP

5

RANDOM_TYPE

add_an_upginb

FILE

renadd05.ped

(CO)VARIANCES

41.967 22.512 24.058 26.907

22.512 17.489 19.738 24.257

24.058 19.738 28.775 34.741

26.907 24.257 34.741 56.668

OPTION SNP_file simsnp.txt

OPTION store_pev_pec 5 full

OPTION solfile solutions_partial

This will produce the following outputs: ebv_pev_1_1, ebv_pev_2_1, ebv_pev_3_1, and ebv_pev_4_1, which should be renamed as ebv_pev_1_1_partial, ebv_pev_2_1_partial, ebv_pev_3_1_partial, ebv_pev_4_1_partial, respectively.

Parameter file for validationf90

# BLUPF90 parameter file created by RENUMF90

DATAFILE

renf90_whole.dat

NUMBER_OF_TRAITS

4

NUMBER_OF_EFFECTS

5

OBSERVATION(S)

1 2 3 4

WEIGHT(S)

EFFECTS: POSITIONS_IN_DATAFILE NUMBER_OF_LEVELS TYPE_OF_EFFECT[EFFECT NESTED]

5 5 5 5 1 cross # Mean

6 6 6 6 155 cross # Year

7 7 7 7 2 cross # Sex

8 8 8 8 11 cross # Year

9 9 9 9 4641 cross # Animal

RANDOM_RESIDUAL VALUES

45.124 22.357 18.626 13.762

22.357 44.210 22.690 18.016

18.626 22.690 46.101 22.795

13.762 18.016 22.795 45.274

RANDOM_GROUP

5

RANDOM_TYPE

add_an_upginb

FILE

renadd05.ped

(CO)VARIANCES

41.967 22.512 24.058 26.907

22.512 17.489 19.738 24.257

24.058 19.738 28.775 34.741

26.907 24.257 34.741 56.668

OPTION SNP_file simsnp.txt

OPTION validation 5 val_set

OPTION predictive_ability

OPTION se exact

which will produce the following output:

valf90.par

VALIDATIONF90 ver. 1.01

Parameter file: valf90.par

Data file: renf90_whole.dat

Number of Traits 4

Number of Effects 5

Position of Observations 1 2 3 4

Position of Weights 0 0 0 0

Value of Missing Trait/Observation 0

EFFECTS

# type position (2) levels [positions for nested]

1 cross-classified 5 5 5 5 1

2 cross-classified 6 6 6 6 155

3 cross-classified 7 7 7 7 2

4 cross-classified 8 8 8 8 11

5 cross-classified 9 9 9 9 4641

Residual (co)variance Matrix

45.124 22.357 18.626 13.762

22.357 44.210 22.690 18.016

18.626 22.690 46.101 22.795

13.762 18.016 22.795 45.274

Random Effect(s) 5

Type of Random Effect: additive animal with unknown parent groups and inbreeding

Pedigree File: renadd05.ped

trait effect (CO)VARIANCES

1 5 41.97 22.51 24.06 26.91

2 5 22.51 17.49 19.74 24.26

3 5 24.06 19.74 28.77 34.74

4 5 26.91 24.26 34.74 56.67

REMARKS

(1) Weight position 0 means no weights utilized

(2) Effect positions of 0 for some effects and traits means that such

effects are missing for specified traits

* Validation for individuals in effect: 5

* File(s) containing validation set ids: val_set

* Calculate predictive ability from yhat_residual

* Solutions read from sol_and_acc_whole and sol_and_acc_partial

* Method to calculate confidence intervals for statistics: exact

Options read from parameter file for genomic

* SNP format: BLUPF90 standard (text)

* SNP file: simsnp.txt

* SNP Xref file: simsnp.txt_XrefID

* ReadGInverse (default=.false.)

Heritability for trait 1: 0.482

Heritability for trait 2: 0.283

Heritability for trait 3: 0.384

Heritability for trait 4: 0.556

Reading and renumbering pedigree

Done

Genetic variance for the focal animals for trait 1: 40.777

Genetic variance for the focal animals for trait 2: 16.993

Genetic variance for the focal animals for trait 3: 27.959

Genetic variance for the focal animals for trait 4: 55.061

*--------------------------------------------------------------*

* Genomic Library: Version 1.316 *

* *

* Optimized OpenMP Version - 32 threads *

* *

* Modified relationship matrix (H) created for effect: 5 *

*--------------------------------------------------------------*

Read 4641 animals from pedigree file: "renadd05.ped"

Number of Genotyped Animals: 1260

Creating A22

Extracting subset of: 2005 pedigrees from: 4641 elapsed time: 0.0079

Calculating A22 Matrix by Colleau OpenMP...elapsed time: .0085

Numbers of threads=16 32

------------------------------

Final Pedigree-Based Matrix

------------------------------

Statistic of Rel. Matrix A22

N Mean Min Max Var

Diagonal 1260 1.023 1.000 1.289 0.002

Off-diagonal 1586340 0.056 0.000 0.812 0.004

Reading G-inverse from file: "Gi"

elapsed time= 0.0

--------------------------

Final Genomic Inv Matrix

--------------------------

Statistic of Inv. Genomic Matrix

N Mean Min Max Var

Diagonal 1260 4.278 2.462 45.068 12.393

Off-diagonal 1586340 -0.003 -4.247 2.922 0.014

*--------------------------------------------------*

* Setup Genomic Done !!!, elapsed time: 0.120 *

*--------------------------------------------------*

Inverse LAPACK MKL dpotrf/i #threads= 16 32 Elapsed omp_get_time: 0.0452

Cross-validation statistics for trait: 1

Value SE L_95%_CI H_95%_CI

Absolute bias: -0.030 0.142 -0.309 0.249

Bias in genetic sd: -0.005 0.022 -0.048 0.038

Dispersion: 0.941 0.056 0.831 1.052

Ratio of accuracies: 0.760 0.000 0.716 0.798

Reliability : 0.389 0.116 0.162 0.617

Predictive ability: 0.491 0.000 0.388 0.630

Cross-validation statistics for trait: 2

Value SE L_95%_CI H_95%_CI

Absolute bias: -0.079 0.088 -0.251 0.093

Bias in genetic sd: -0.019 0.021 -0.060 0.022

Dispersion: 0.863 0.057 0.751 0.974

Ratio of accuracies: 0.729 0.000 0.680 0.771

Reliability : 0.361 0.115 0.136 0.586

Predictive ability: 0.242 0.000 0.066 0.421

Cross-validation statistics for trait: 3

Value SE L_95%_CI H_95%_CI

Absolute bias: -0.064 0.114 -0.287 0.158

Bias in genetic sd: -0.012 0.021 -0.054 0.030

Dispersion: 0.864 0.056 0.754 0.975

Ratio of accuracies: 0.741 0.000 0.695 0.782

Reliability : 0.363 0.116 0.136 0.589

Predictive ability: 0.337 0.000 0.195 0.491

Cross-validation statistics for trait: 4

Value SE L_95%_CI H_95%_CI

Absolute bias: -0.034 0.164 -0.355 0.288

Bias in genetic sd: -0.004 0.022 -0.047 0.038

Dispersion: 0.959 0.054 0.853 1.065

Ratio of accuracies: 0.788 0.000 0.749 0.822

Reliability : 0.405 0.117 0.176 0.633

Predictive ability: 0.537 0.000 0.452 0.665
